# Antibiotic resistance genes of public health importance in livestock and humans in an informal urban community in Nepal

**DOI:** 10.1038/s41598-022-14781-y

**Published:** 2022-08-15

**Authors:** Cristin C. W. Young, Dibesh Karmacharya, Manisha Bista, Ajay N. Sharma, Tracey Goldstein, Jonna A. K. Mazet, Christine K. Johnson

**Affiliations:** 1 EpiCenter for Disease Dynamics, School of Veterinary Medicine, University of California, Davis, 1089 Veterinary Medicine Drive, Davis, CA 95616 USA; 2 Center for Molecular Dynamics, Nepal (CMDN), Thapathali Road 11, Kathmandu, 44600 Nepal; 3 Mott MacDonald, Noida, Uttar Pradesh India; 4 One Health Institute, School of Veterinary Medicine, University of California, Davis, CA USA

**Keywords:** Epidemiology, Bacterial infection

## Abstract

Efforts to mitigate the increasing emergence of antimicrobial resistance (AMR) will benefit from a One Health perspective, as over half of animal antimicrobials are also considered medically important in humans, and AMR can be maintained in the environment. This is especially pertinent to low- and middle-income countries and in community settings, where an estimated 80% of all antibiotics are used. This study features AMR genes found among humans, animals, and water at an urban informal settlement in Nepal with intensifying livestock production. We sampled humans, chickens, ducks, swine, and water clustered by household, as well as rodents and shrews near dwellings, concurrently in time in July 2017 in southeastern Kathmandu along the Manohara river. Real-time qualitative PCR was performed to screen for 88 genes. Our results characterize the animal-human-environmental interfaces related to the occurrence of specific resistance genes (*bla*_SHV-1_ (SHV(238G240E) strain), *QnrS*, *ermC*, *tetA*, *tetB, aacC2*, *aadA1*) associated with antibiotics of global health importance that comprise several drug classes, including aminoglycosides, beta-lactams, tetracyclines, macrolides, and fluoroquinolones. By characterizing risk factors across AMR genes of public health importance, this research highlights potential transmission pathways for further investigation and provides prioritization of community-based prevention and intervention efforts for disrupting AMR transmission of critically important antibiotics used in both humans and animals in Nepal.

## Introduction

Antimicrobial resistance (AMR) is a pressing global health threat recognized by international organizations worldwide^1,2^. Recent reports from the World Health Organization (WHO) and the World Organisation for Animal Health (OIE) have called for further research to identify and characterize risk factors of antimicrobial resistance genes of public health importance^1^. In 2017, the WHO published a list of priority pathogens that are antibiotic resistant, comprising 12 bacterial families in three prioritization categories that the organization has highlighted as those posing the greatest threat to human health worldwide^1^. The highest priority category (critical) includes multidrug resistant bacteria such as *Acinetobacter, Pseudomonas*, and several Enterobacteriaceae (e.g., *Klebsiella* and *Escherichia coli*)^1^. Additionally, in partnership with the Food and Agriculture Organization of the United Nations (FAO) and the OIE, the WHO published a list of critically important antimicrobials to humans and animals in 2005, with the latest update in 2016^3^. The OIE has also published a report on the use of antimicrobials in animals^4^. Both reports were published with the recognition that AMR selection pressures should be minimized or prevented for both human and non-human pathogens via careful consideration of antibiotic use and authorization in both populations. Moreover, the reports were made public and are continually updated with the goals of showing where there is overlap in the two lists, of giving guidance on resource allocation for new and existing drug applications, estimating harm to people, and developing policies and interventions for restricted use in a country^3^.

As over 60% of antimicrobials approved for use in 2015 in animals are also considered medically important in humans^5^, a One Health approach is critically important for characterizing potential reservoirs of AMR and identifying risk factors that could elucidate routes of AMR transmission and carriage of resistance genes across humans, animals, and the environment. While prior studies have assessed global drivers of the emergence and transmission of AMR^6–8^, research is needed with a focus on modeling predictors for the presence of resistance genes from a one health perspective across human, animal, and environmental populations. This is especially true in low- and middle-income countries, which saw the greatest increase of per capita human consumption of antimicrobials between 2000–2010 compared to high-income countries, and where there are poor diagnostics, inadequate supplies of antimicrobials, and high population densities coupled with poor sewage system infrastructure^6,9^. Additionally, while hospital usage of antibiotics is an important aspect of AMR transmission, an estimated 80% of all antibiotics are used in a community setting^9^. While prior studies have explored AMR transmission in community settings in low- and middle-income countries^10,11^, research has focused on one at-risk population (e.g., children)^12–14^, on one bacterium or drug class (e.g., *E. coli*)^15,16^, or on one route of transmission (e.g., sewage effluent)^17–19^, which may not accurately represent the underlying mechanisms of the entire antibiotic resistome in these settings. Surveillance of AMR across a larger range of bacteria and resistance genes would aid in characterizing the phenotypic and genotypic resistome, while sampling from a One Health perspective across humans, animals, and their environment would provide insights into where reservoirs of resistance might exist in a community. To our knowledge, no studies have focused on multiple resistance genes across a broad range of drug classes while sampling from all parts of the community in a low-income or middle-income country such as Nepal. Furthermore, community-based research in Nepal is lacking, especially in informal settlement populations such as those that expanded rapidly in Kathmandu after the 2015 earthquake^20,21^.

To address this gap in the literature, our prior research characterized the community resistome of an urban informal settlement study site with intensifying livestock production within Nepal (Young et al., *manuscript submitted*). For this study, we evaluated risk factors relating to the occurrence of resistance genes associated with antibiotic-resistant bacteria of global health importance for both humans and animals and chose to focus on a subset of seven genes (*bla*_SHV-1_ (SHV(238G240E) strain), *QnrS*, *ermC*, *tetA*, *tetB, aacC2*, and *aadA1*) that encompass a broad range of drug classes, including aminoglycosides, beta-lactams, tetracyclines, macrolides, and fluoroquinolones. Results from this research can inform AMR surveillance and policies in Nepal in both the human and agricultural sectors by providing an understanding of the resistance gene burden and thus the background risk for AMR spread in the broader community, thus allowing for the prioritization of where to focus further research and intervention efforts.

## Results

Sixty-nine out of 88 resistance genes were detected in 13 out of 16 antibiotic classification groups and in all but 12 samples (7 rodent, 5 water samples). All seven genes prioritized for this analysis (*tetA, tetB, aadA1, QnrS, bla*_SHV-1_, *ermC*, and *aacC2*) were detected in all sources sampled with the exception of rodents (*Rattus spp.).* In addition, *ermC* was not detected in humans sampled in this study (Table 1, Fig. 2G). Only *tetA* and *aadA1* were found in river samples, while all seven genes were found in well samples (Table 1). The majority of cloacal swab-only samples were positive for *tetA* (100% of samples positive), *QnrS* (80%), *ermC* (70%), *tetB* (80%), and *aadA1* (90%), and the majority of human fecal samples had detectable amounts of *bla*_SHV-1_ (82% of samples positive), *QnrS* (91%), and *tetA* (91%) resistance genes (Table 1).

**Table 1:** Specimens positive for seven antibiotic resistance genes of public health importance in an urban informal settlement in Kathmandu, Nepal, 2017.

	n (%)						
	*bla*_*SHV-1*_	*QnrS*	*ermC*	*tetA*	*tetB*	*aacC2*	*aadA1*
**Sample type**							
Fecal sample^a^ and oral swab	7 (32)	18 (62)	23 (50)	33 (92)	41 (95)	17 (46)	22 (63)
Cloacal swab	4 (31)	16 (89)	12 (80)	12 (100)	8 (80)	13 (72)	18 (95)
Fecal sample	25 (93)	24 (96)	0 (0)	27 (96)	9 (69)	8 (53)	14 (74)
Rectal swab	8 (20)	9 (29)	2 (6)	17 (55)	11 (35)	10 (32)	13 (42)
Oral swab	19 (37)	6 (15)	6 (15)	7 (19)	34 (72)	8 (19)	5 (13)
Jar of water	0 (0)	0 (0)	0 (0)	0 (0)	0 (0)	0 (0)	0 (0)
River water	0 (0)	0 (0)	0 (0)	1 (33)	0 (0)	0 (0)	1 (3)
Well water	3 (23)	2 (15)	5 (38)	7 (54)	2 (15)	3 (23)	7 (54)
**Sample source**							
Chicken	12 (44)	23 (85)	23 (85)	27 (100)	26 (96)	23 (85)	27 (100)
Duck	14 (74)	17 (89)	18 (95)	19 (100)	18 (95)	15 (79)	18 (95)
Human	29 (43)	24 (36)	0 (0)	33 (49)	48 (72)	8 (12)	14 (21)
Rodent	0 (0)	0 (0)	0 (0)	0 (0)	0 (0)	0 (0)	0 (0)
Shrew	4 (33)	5 (42)	1 (8.3)	7 (58)	4 (33)	3 (25)	5 (42)
Swine	4 (36)	4 (36)	1 (9.1)	10 (91)	7 (64)	7 (64)	8 (73)
Water	3 (18)	2 (12)	5 (29)	8 (47)	2 (12)	3 (18)	8 (47)

A total of 218 specimens from 161 individuals were tested, including: humans (n = 88), chickens (n = 47), ducks (n = 35), swine (n = 11), water (n = 17), rodents (n = 8), and shrews (n = 12). A subset of data (n = 161) was used in analyses, where within-individual samples were collapsed and counted as positive for both oral and fecal sampling if both of the two samples (fecal or oral) were positive for a particular resistance gene, and otherwise categorized according to whether the oral or fecal sample was positive.

^a^Fecal samples comprise rectal swabs (swine, rodents, shrews), cloacal swabs (ducks, chickens), and feces (humans).

### Gene-specific Analyses

#### *bla*_*SHV-1*_ antibiotic resistance gene

**Figure 1:**
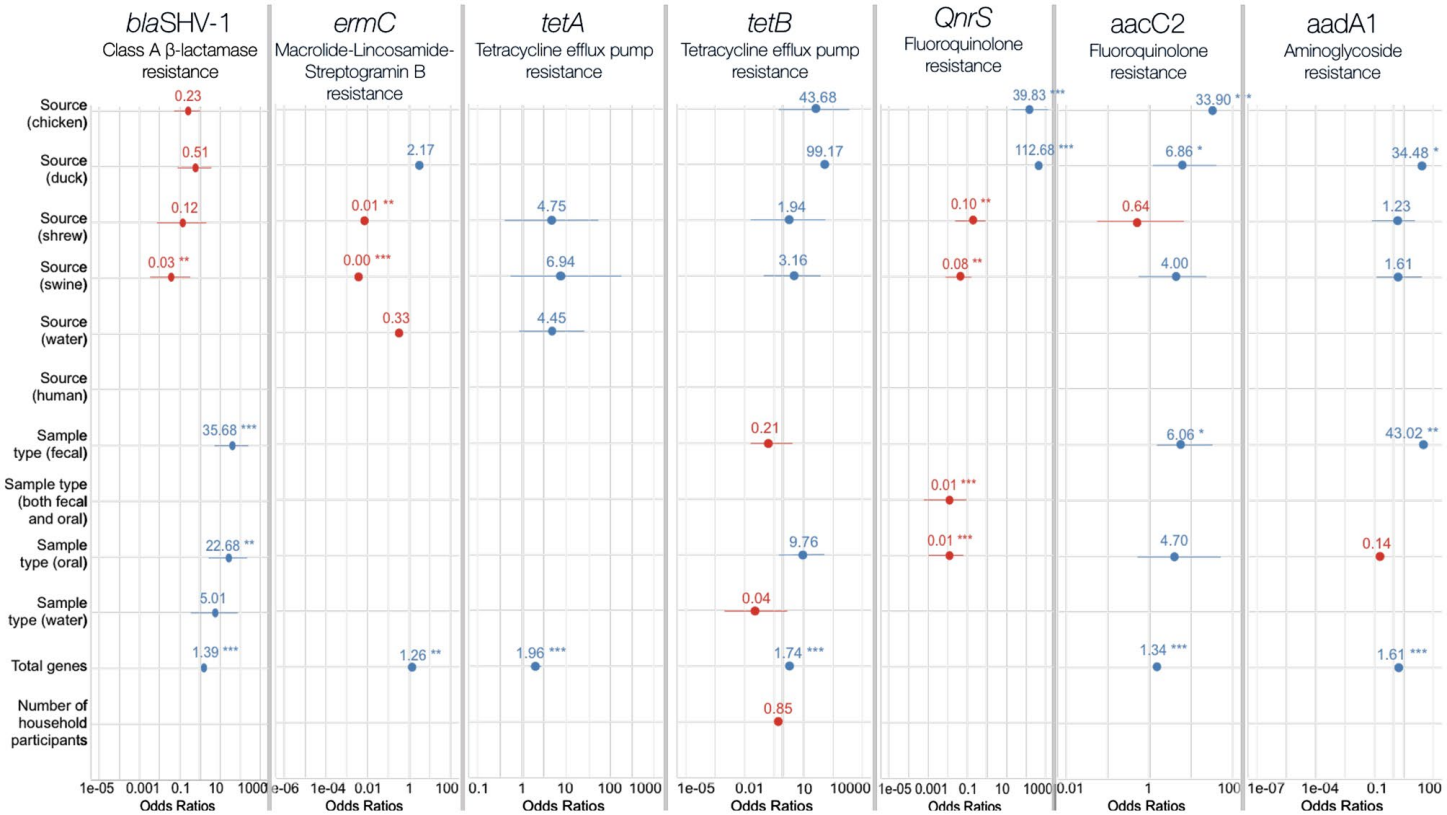
Logistic regression output of predictors associated with the detection of antibiotic resistance genes of public health importance in an urban informal settlement in Kathmandu, Nepal, 2017. Odds ratios and 95% confidence intervals indicated by point and line for each variable across models; specific results can be found in SI Table 1. Asterisks denote p-values (*p < 0.05, **p < 0.001, ***p < 0.0001). Colors indicate a protective (red) or risk (blue) factor (OR < 1 vs. > 1). Odds ratio scales shown at the bottom of each graph and vary by model. Not all variables were assessed in each model; see “Methods” section for specifics. *bla*_*SHV-1*_ and *tetB* were mixed effects models and only fixed effect output is shown. Fecal samples comprise rectal swabs (swine, rodents, shrews), cloacal swabs (ducks, chickens), and feces (humans). Total genes serves as a proxy for co-occurrence of genes in the sample, as measured by total number of genes (out of N = 88) positive in the sample.

**Figure 2:**
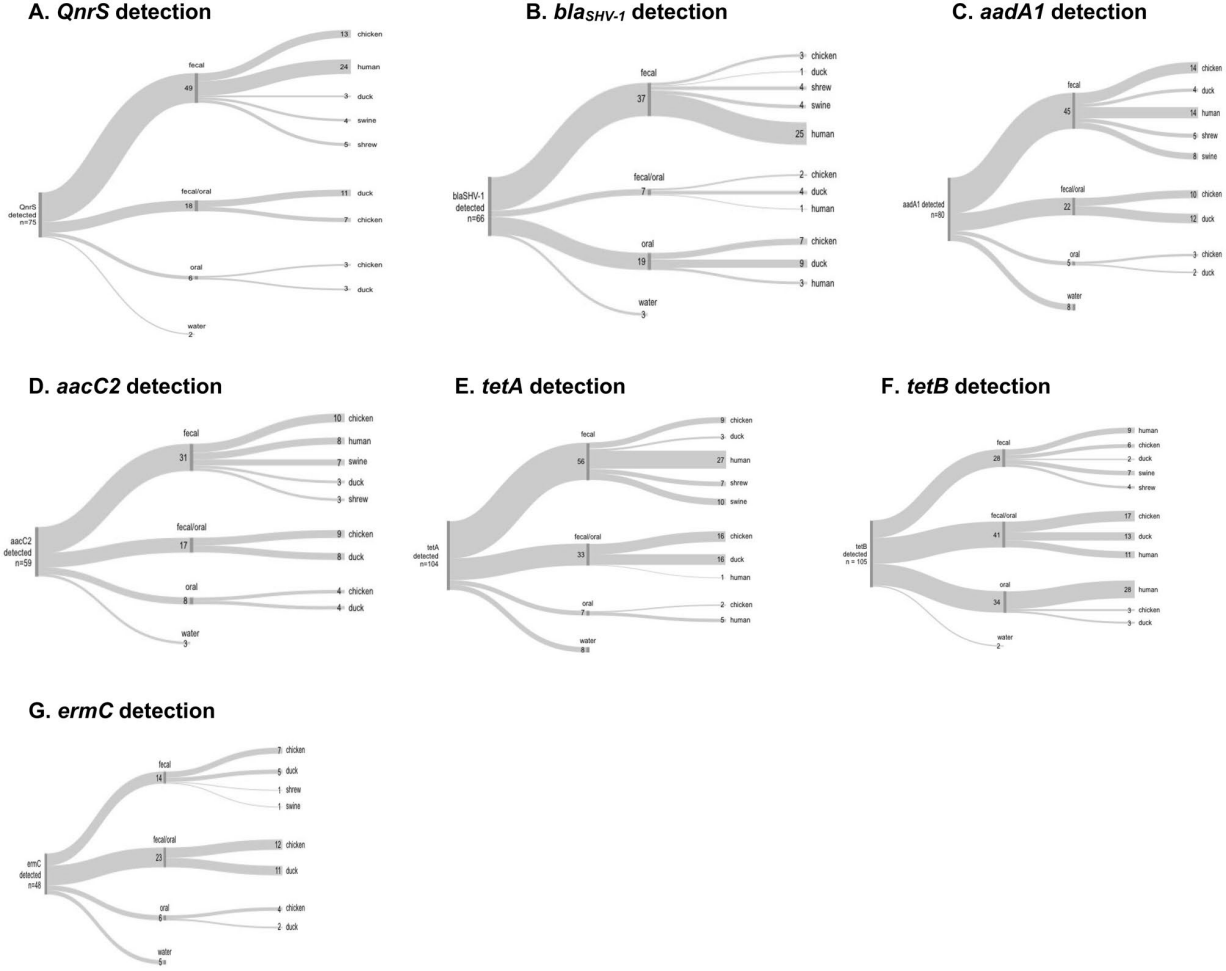
(**A–G**) Flowcharts of positive specimens from an urban, informal settlement in Kathmandu, Nepal separated by sample type and source sampled across seven antibiotic resistance genes of public health importance. Alluvial diagrams of positive specimens across seven antibiotic resistance genes of public health importance. Flow diagram should be read from left to right, with total numbers of samples positive on the left, sample type (fecal only, fecal and oral, oral only, or water) in the middle, and source sampled (chicken, duck, human, shrew, swine, water, or rodent) on the right. Numbers indicate number of samples positive for the AMR gene specified.

Across sample types, *bla*_SHV-1_ was most prevalent in human fecal samples (92.6% detection in stool specimens, Fig. 2B). This gene was also commonly detected in water samples (21.4% positive), fecal samples from animals (30.8% detection in cloacal swabs, 25.8% detection in rectal swabs), or oral swabs (37.3% positive). Of the 43.3% of human samples in which *bla*_SHV-1_ was detected, stool specimens from animal sources accounted for the majority of positive samples. Over 70% of ducks sampled (n = 14) were positive for *bla*_SHV-1_, with 64% of detection in oral samples versus cloacal swabs. This *bla*_SHV-1_ variant was also found in chickens (44.4%), shrews (33.3%), swine (36.4%), and water (21.4%). In the multivariate model (fixed effects pseudo-R^2^= 0.63), there were lower odds of finding *bla*_SHV-1_ in ducks and swine compared to humans (reference category, Fig. 1, Table 2). Co-occurrence of other resistance genes in the specimen sampled was found to be a predictor for detection of *bla*_SHV-1_; for every additional gene found in the sampled specimen, there were 1.39 (p < 0.0001) times higher odds of being positive for this *bla*_SHV-1_ gene variant. Additionally, there were over 20 times the odds of detecting *bla*_SHV-1_ in either oral swabs (p = 0.005) or fecal samples (p < 0.0001) compared to finding the gene in both fecal and oral samples from an individual (Fig. 1, Table 2). Fecal samples were associated with a higher odds of detection of *bla*_SHV-1_, suggesting that this gene is predominantly shed in the feces of people and animals.

**Table 2:** Logistic regression analysis of predictors associated with the detection of antibiotic resistance genes of public health importance in an urban informal settlement in Kathmandu, Nepal, 2017.

Predictor variable	Odds ratio	95% Confidence Interval	p value
*bla*_SHV-1*_			
**Sample type**			
Fecal sample** and oral swab	Reference	-	–
Fecal sample	**35.68**	**(5.18, 245.58)**	**< 0.0001**
Oral swab	**22.68**	**(2.51, 205.08)**	**0.005**
Water	5.01	(0.34, 73.61)	0.240
Co-occurrence of genes in sample***	**1.39**	**(1.21, 1.59)**	**< 0.0001**
**Sample source**			
Human	Reference	–	–
Chicken	0.23	(0.05, 1.02)	0.053
Duck	**0.51**	**(0.07, 3.60)**	0.502
Shrew	0.12	(0.007, 2.05)	0.144
Swine	**0.03**	**(0.003, 0.32)**	**0.003**

*QnrS*			
**Sample type**			
Fecal sample and oral swab	**0.008**	**(0.001, 0.08)**	**< 0.0001**
Fecal sample	Reference	-	–
Oral swab	**0.006**	**(0.001, 0.04)**	**< 0.001**
**Sample source**			
Human	Reference	-	–
Chicken	**39.83**	**(5.08, 312.29)**	**< 0.001**
Duck	**112.68**	**(11.49, 1105.46)**	**< 0.001**
Shrew	**0.10**	**(0.018, 0.50)**	**0.006**
Swine	**0.08**	**(0.014, 0.43)**	**0.003**

*ermC*			
**Co-occurrence of genes in sample**	**1.26**	**(1.08, 1.46)**	**0.003**
**Sample source**			
Chicken	Reference	–	–
Duck	2.17	(0.18, 25.61)	0.538
Shrew	**0.008**	**(0.004, 0.15)**	**0.001**
Swine	**0.005**	**(0.002, 0.09)**	**< 0.0001**
Water	0.33	(0.05, 2.23)	0.256

*tetA*			
**Co-occurrence of genes in sample**	**1.96**	**(1.51, 2.54)**	**< 0.0001**
**Sample source**			
Human	Reference	–	–
Shrew	4.75	(0.46, 49.17)	0.192
Swine	6.94	(0.49, 98.17)	0.152
Water	4.45	(0.82, 24.26)	0.084

tetB*			
**Sample type**			
Fecal sample and oral swab	Reference	–	–
Fecal sample	0.24	(0.023, 2.45)	0.229
Oral swab	7.95	(0.72, 87.57)	0.090
Water	0.05	(0.001, 1.58)	0.088
**Co-occurrence of genes in sample**	**1.65**	**(1.29, 2.11)**	**< 0.0001**
**Sample source**			
Human	Reference	–	–
Chicken	**28.44**	**(1.04, 775.77)**	**0.047**
Duck	**56.97**	**(1.20, 2712.08)**	**0.040**
Shrew	1.63	(0.02, 138.43)	0.829
Swine	2.65	(0.11, 65.81)	0.553
**Number of people in household**	0.87	(0.76, 1.002)	0.053

*aacC2 *			
**Sample type**			
Fecal sample and oral swab	Reference	–	–
Fecal sample	**6.06**	**(1.20, 30.63)**	**0.029**
Oral swab	4.70	(0.43, 51.66)	0.205
**Co-occurrence of genes in sample**	**1.34**	**(1.18, 1.52)**	**< 0.0001**
**Sample source**			
Human	Reference	–	–
Chicken	**33.90**	**(6.54, 175.85)**	**< 0.0001**
Duck	**6.86**	**(1.12, 42.13)**	**0.038**
Shrew	0.64	(0.06, 6.56)	0.704
Swine	4.00	(0.54, 29.59)	0.175

*aadA1*			
**Sample type**			
Fecal sample and oral swab	Reference	–	–
Fecal sample	**43.02**	**(2.86, 646.30)**	**0.007**
Oral swab	0.15	(0.001, 119.96)	0.750
**Co-occurrence of genes in sample**	**1.61**		**0.001**
**Sample source**			
Human	Reference	–	–
Duck	**34.48**	**(1.71, 696.93)**	**0.021**
Shrew	1.24	(0.08, 19.003)	0.880
Swine	1.61	(0.11, 23.08)	0.725

Bolded rows show outcomes with p < 0.05.

*Mixed effects models; only fixed effect output is shown.

**Fecal samples comprise rectal swabs (swine, rodents, shrews), cloacal swabs (ducks, chickens), and feces (humans).

***As measured by total number of genes (out of n = 88) positive in the sample.

#### QnrS antibiotic resistance gene

The majority of poultry sampled had detectable levels of *QnrS* (chickens 85.2%, ducks 89.5%, Fig. 2A). Of note, *QnrS* was found in cloacal swabs in chickens (56.5%), whereas the majority of both oral and cloacal swabs were positive for the gene in ducks (64.7%). Of the 35.8% of human samples that were positive for *QnrS*, all were found only in stool specimens (n = 24). Finally, only 11.8% of water samples were positive for *QnrS*. Co-occurrence of genes in the sample was dropped from the full model for *QnrS* due to high positive correlation with both the source sampled and sample type variables (McFadden’s pseudo-R^2^ = 0.50). The water categorical level was also excluded from both predictor variables (sample type and source sampled) due to perfect collinearity. *QnrS* was significantly more likely to be detected in fecal samples only (Fig. 1, Table 2). Additionally, chickens and ducks had significantly higher odds of having a sample with *QnrS* compared to humans (the reference group). In contrast, shrews were over 10 times less likely to have *QnrS* detected than humans (p = 0.006) and swine had a 13-fold lower odds of having *QnrS* detected than humans (p = 0.003, Fig. 1, Table 2).

#### ermC antibiotic resistance gene

Chickens and ducks were the main sources of *ermC* in this community, with 85.2% and 94.7% detection rates in each species, respectively (Fig. 2G). The gene was not detected in humans or rodents, and less commonly in shrews (8.3%), swine (9.1%), or water (29.4%). Both ducks and chickens had *ermC* broadly detected in both oral and cloacal swabs (61.1% and 52.2% of positive samples, respectively), with *ermC* more often detected in cloacal swabs or both samples than in oral swabs alone (14.6% prevalence in oral swabs only). Due to the absence of detection in rodent and human samples, both were dropped from the multivariate model. In the multivariate model (McFadden’s pseudo-R^2^ = 0.68), chickens had significantly higher odds of having a sample positive for *ermC* compared to both shrew and swine samples (Fig. 1, Table 2). In addition, co-occurrence of other resistance genes in the sample was associated with a 26% increase in detection of *ermC* (p = 0.003, Fig. 1, Table 2). Sample type and the number of participants in a household were not significantly associated with the presence or absence of *ermC* in a specimen sampled in the final model.

#### tetA antibiotic resistance gene

Resistance gene *TetA* was detected in every chicken and duck sample in our study (n = 27 and n = 19, respectively, Fig. 2E). Swine were also frequent carriers of *tetA*, as this gene was found in 10 out of 11 swine samples. In humans, *tetA* was primarily detected in fecal samples versus oral swabs, with 96.4% of stool specimens positive compared with 14.3% of oral swabs. In contrast, the majority of chickens and ducks had *tetA* detected in both cloacal and oral swabs (59.3% and 84.2%, respectively). Finally, just over half of shrews had detectable levels of *tetA* (58.3%), and just under half of water samples were positive (47.1%), including one river sample. In multivariate modeling, chickens and ducks were dropped from the multivariate model as *tetA* was detected in every sample from these two species. Sample type was not a significant predictor of *tetA* presence in a specimen sampled in the final model (McFadden’s pseudo-R^2^ = 0.57). However, as with the previous gene-specific models, co-occurrence of genes in a sample was significantly associated with finding *tetA* in the sample (OR 1.96, p < 0.0001, Fig. 1, Table 2). Possible explanations for this include *tetA* co-occurring with other genes sampled in the full array of 88 resistance genes, or external factors (e.g., water/sanitation/hygiene sources or antibiotic usage) contributing to AMR gene selection in the individual.

#### tetB antibiotic resistance gene

As with *tetA*, *tetB* was highly prevalent in chickens and ducks sampled in this community (96.3% and 94.7% positive, Fig. 2F). Over 70% of humans sampled were also positive. The hierarchical mixed effects model, with household ID as a random effect, indicated household clustering of tetB (Table 2). This was the only AMR gene for which clustering within households was evident. Water samples had lower rates of *tetB* compared with *tetA* (11.8% vs. 47.1%), and *tetB* was only detected in well samples. Over 60% of swine carried *tetB* (63.6%), while a third of wild shrews sampled at the study site were carriers for *tetB* (33.3%). All oral swabs tested for chickens and ducks detected *tetB*, and the majority of *tetB* was found in both cloacal and oral swabs for both chickens (65.4%) and ducks (72.2%). In contrast to *tetA* detection, which was predominantly found in fecal samples in both humans and animals, the majority of *tetB* detected was in oral samples in humans (58.3%). In the final model for *tetB* (fixed effects pseudo-R^2^ = 0.87), co-occurrence of genes sampled was significantly associated with detection of *tetB*; for every additional resistance gene found in the specimen sampled, there were 1.65 times the odds of finding *tetB* in the sample (p < 0.0001, Fig. 1, Table 2). Both ducks and chickens had significantly higher odds of having *tetB* in a sample compared with humans (Fig. 1, Table 2).

#### aacC2 antibiotic resistance gene

The *aacC2* gene was less common in humans in this community (11.9% prevalence) compared to chickens (85.2% prevalence) and ducks (78.9% prevalence, Fig. 2D). The gene was detected in a quarter of shrews sampled, over half of swine sampled (58.3%) and in only three water samples (17.6%, from wells). Compared with fecal samples, *aacC2* was less frequently detected in oral swabs (18.6%). No oral swabs from humans were positive for *aacC2*. In chickens, *aacC2* was frequently detected in cloacal swabs (43.5%) as well as in both samples in individuals sampled both cloacally and orally (39.1%). In comparison, *aacC2* was primarily found in both cloacal and oral swabs of ducks, (53.3%). In the multivariate model for *aacC2* (McFadden’s pseudo-R^2^ = 0.60), there was a higher odds of finding *aacC2* in chickens and ducks compared to humans (the reference category, Fig. 1, Table 2). Compared with finding the gene in both a fecal and an oral sample, *aacC2* was over six times more likely to be found in a fecal sample only (p = 0.029). Finally, *aacC2* was 1.34 times more likely to be found with other AMR genes in a sample (p < 0.0001).

#### aadA1 antibiotic resistance gene

Chickens and ducks had high prevalences of *aadA1* in this community (100% and 94.7%, respectively), with the gene primarily detected in cloacal swabs (94.7%) or both cloacal and oral swabs (62.9%) compared to oral swabs only (12.5%, Fig. 2C). In contrast, 20.9% of humans sampled were positive for *aadA1*, with 100% of positive samples being in stool specimens. Two-thirds of swine were carriers for *aadA1*, while under half of both shrew samples (41.7%) and water samples (47.1%) were positive, including one river sample. Sample type, co-occurrence of genes in the sample, and sample source were all significant predictors of finding *aadA1* in a specimen in the final model (McFadden’s pseudo-R^2^ = 0.77, Fig. 1, Table 2). For every additional resistance gene found in a sample, there were 1.61 times the odds of finding *aadA1* (p = 0.001). There was a significantly higher odds of finding *aadA1* in ducks compared to humans (the reference group); *aadA1* was found in every chicken sampled and so this category was not included in the final model. Across sample types, *aadA1* was 43 times more likely to be found in fecal samples only than in both fecal and oral samples (p = 0.007). Water was excluded as a category of sample type due to perfect collinearity with source sampled in the final model.

#### Comparisons across all gene models

For all genes modeled except *QnrS*, co-occurrence of genes in the sample tested was positively associated with finding the gene in question, with an increase in odds ranging from 1.26 to 1.96 across models (Fig. 1, Table 2), holding all other factors constant. There were higher odds of finding *bla*_SHV-1_ in humans than all other sources sampled, holding all other factors constant. In contrast, there were lower odds of finding, *tetA*, *tetB*, *aacC2*, and *aadA1* in humans than in all other sources sampled. This was true of *QnrS* as well for chickens and ducks, whereas *QnrS* was less likely to be found in shrews and swine compared to humans. Chickens were far more likely to have *ermC* detection than shrews or swine, holding all other factors constant. Sample type was a predictor of finding *bla*_SHV-1_, *QnrS*, *aacC2*, and *aadA1*; all were significantly more likely to be found in fecal samples only compared with both fecal and oral samples, holding all other factors constant. With the exception of *bla*_SHV-1_, all resistance genes assessed in this analysis were more likely to be found in poultry and in fecal samples, indicating that poultry as well as fecal contamination in this community are likely sources of resistance to antibiotics used to treat bacterial pathogens of public health importance in Nepal.

## Discussion

Our results highlight risk factors related to the occurrence of seven resistance genes (*bla*_SHV-1_ (SHV(238G240E) strain), *QnrS*, *ermC*, *tetA*, *tetB, aacC2*, and *aadA1*) associated with antibiotic-resistant bacteria of public health importance in samples from humans, animals, and the environment in an urban informal settlement community setting in Kathmandu, Nepal. Genes chosen for this analysis encompass resistance to a broad range of drug classes, including aminoglycosides, beta-lactams, tetracyclines, macrolides, and fluoroquinolones. All seven genes in this analysis were detected across all species and sources sampled in this study, with the exception of *Rattus spp.* samples, for which none of the genes were detected (Fig. 2). In multivariate analyses, factors for detection of these genes differed across genes and resistance classes. This study focused on associations related to detection across the seven genes in order to generate hypotheses for potential transmission pathways in an urban, informal settlement community setting.

By assessing which species and sample types were associated with detection of a gene in our study, we can begin to explore the complex transmission routes of AMR in the broader community and thus prioritize where to focus further research and prevention/intervention efforts. Prior studies have assessed indicators for carriage of AMR genes in agricultural settings. For instance, in research conducted on livestock farms in Vietnam, *E. coli* was found across chicken, duck, and pig farms, as well as in wild small mammals including *Rattus spp.* and *Suncus murinus* shrews^22^. Additionally, Nhung et al. (2015) found that pig farms, the size of the farm, and well water were significantly associated with multidrug resistance in *E. coli*^22^. In a 2017 study investigating the role of mobile genetic elements in the spread of resistant *E. coli* from chickens to humans in rural Ecuador, Moser et al. found that isolates from small-scale chicken operations contributed to higher levels of resistance in the community sampled via selection for isolates carrying mobile genetic elements^23^.

To our knowledge, this is the first study to date to have concurrently sampled across an entire community and across a broad range of antibiotic resistance genes. Additionally, our study is the first to show that while associations with specific risk factors were not uniform across resistance genes tested, poultry (chickens and ducks) and fecal samples were indicators for detection of six out of seven of the genes investigated here, suggesting that surveillance and intervention efforts should focus on poultry and feces for optimal detection and reduction of these resistance genes in this community. We also present results of novel detection of several AMR genes in previously unstudied wild small mammal species, including detection of all seven genes in *Crocidura lasiura*, and detection of six of the seven genes (excluding *ermC*) in *Suncus murinus*. The detection of these genes of public health importance in multiple wild small mammal species underscores the need for further research into wildlife reservoirs of antibiotic resistance.

### Risk factors for detection of resistance genes of importance

In our analyses, both pairs of genes belonging to the same antibiotic classification groups (*tetA*/*tetB* and *aacC2*/*aadA1*) had similar predictors for detection. For the genes in the tetracycline efflux pump resistance group (*tetA* and *tetB*), poultry were at high risk of carrying both genes. All chickens and ducks sampled in our study were positive for *tetA*, and so were excluded from the *tetA* regression model; chickens and ducks were significantly more likely to have *tetB* in our modeling results (Fig. 1, Table 2). Because of the high rates of *tetA* and *tetB* in poultry in this community, use of antibiotics in this drug class (e.g., tetracycline or doxycycline) should be limited to only where absolutely necessary, in order to limit the spread of tetracycline efflux pump resistance among poultry and from poultry into the environment or to humans. The second grouping of genes in our study, *aacC2* and *aadA1* (both conferring aminoglycoside resistance), were also similar in their risk factors for detection. Both were more likely to be detected in chickens and ducks and had high odds of being found in fecal samples (Fig. 1, Table 2). As with tetracycline resistance, to limit the potential for AMR gene transmission among poultry and between sources sampled, limiting the usage of aminoglycoside antibiotics and limiting exposure to chicken feces might be beneficial in limiting transmission of AMR genes in this community.

For genes that were more likely to be found in humans than other species (*bla*_SHV-1_ and *QnrS*), the possibility exists that beta-lactamase and fluoroquinolone resistance is actively circulating in commensal and/or pathogenic bacteria in the human population, and participants should be tested further to assess the magnitude of AMR risk based on carriage of these genes. Interestingly, AMR genes did not cluster by household except for tetB, so household was not a strong factor related to detection of the same resistance genes. This is likely because AMR genes found in humans were widespread and apparently shared across the community, regardless of household. Animals and humans lived in close quarters in this community with minimal boundaries, even for owned animals. Chickens and pigs were located in separate pens adjacent to the housing structure to which they belonged. Ducks were marked by their owners and kept penned for part of the day, but also allowed to roam free at other times. Close contact and occasional co-mingling between animals and humans is a likely reason why the household affect was not significant in regression modeling, and clustering of genes by household was not detected for most AMR genes. Improved animal biosecurity, sanitation and hygiene practices would help prevent the spread of resistance genes via fecal transmission, especially for *QnrS* and *bla*_SHV-1_, which were both more likely to be found in fecal samples than oral swabs (Fig. 1, Table 2). Examples of hygiene interventions include human behavioral changes such as hand-washing routines after contact with animals and before food preparation. Potential sanitation interventions could focus on implementing community-wide sewage infrastructure (where there currently is none) and upgrading toilets to latrines with drainage.

Lastly, while shrews were carriers of resistance genes in this community, they had a statistically lower odds of having several of the AMR genes tested compared to humans (Fig. 1, Table 2). Furthermore, rodents (*Rattus spp.*) were not carriers of any of the seven resistance genes tested for in this community. Further testing should be done on a larger sample of small wild mammals including shrews and rodents to more fully characterize this population as a reservoir of resistance and to assess the extent of risk wild small mammals might pose as potential transmission sources of resistance genes.

### Prevalences of resistance genes among species and environmental sources sampled

*Qnr* genes have been found across a wide distribution of animals and habitats, including in aquatic and waterborne organisms, suggesting an origin in the natural environment^24–26^. The high prevalence of *QnrS* in chickens and ducks sampled in our study supports this, although further research is needed to evaluate where the genes resided (e.g., in commensal or pathogenic bacteria, or as part of a mobile genetic element). We also found *QnrS* in human, shrew, swine, and water samples, although the prevalence for each source sampled was lower than it was in poultry (36%, 41%, 36%, and 12%, respectively). The only source sampled in which we did not find *QnrS* were rodents.

Of note, *ermC* was not found in any human samples, and in only one shrew and swine sample. It was found overwhelmingly in chicken and duck samples (in 85% and 95% of samples, respectively), perhaps due to the frequent use of macrolides such as erythromycin (SI Table 1) to treat *Staphylococcus aureus* infections in poultry^27^. Previous studies have found similar patterns of high resistance to erythromycin and other macrolides in poultry^28–30^ and that *ermC-*related erythromycin resistance is transferable between poultry and to human isolates of *Staphylococcus aureus*^31^. In addition to macrolides, all but one chicken and one duck sample also carried tetracycline resistance (via *tetA* and *tetB*). This was to be expected, as tetracyclines are the most widely used antibiotics in poultry due to their wide margin of safety and broad spectrum of activity against both gram-negative and gram-positive bacteria as well as Mycoplasma bacteria^27,30^. The poultry sampled in this study also had high rates of resistance to aminoglycosides (*aacC2*: > 79% for both species, *aadA1*: > 95% for both species), especially as compared to human samples (*aacC2*: 12% positive, *aadA1*: 21% positive). It is unclear why aminoglycoside resistance was low in the human population in our results, as both genes have been found in humans in previous studies^32–34^. However, prior studies have found aminoglycoside resistance genes such as *aadA1* and *aacC2* in *E. coli* of animal origin, whereas different aminoglycoside resistance genes (e.g. *aac(6’)-lb-cr*) were more related to human *E. coli* isolates^24,35^. Lastly, *bla*_SHV-1_ was found in less than 50% of all sources sampled except for ducks, where it was positive in 14/19 samples. Despite the low prevalence across most sources sampled in this study, these results support the previous literature, in which ESBL-producing β-lactamases such as the SHV enzymes have been found across humans, animals, and wildlife sources^36,37^.

### Importance in veterinary medicine in Nepal

Despite only testing for resistance genes, the seven resistance genes assessed in this study belong to a broad range of antibiotic resistance classes and are associated with specific drug resistance classes that comprise common antibiotics currently in use in Nepal in animal and human populations^38^. For example, *aadA1* and *aacC2* confer resistance to aminoglycosides such as streptomycin and gentamicin, both of which are categorized as extremely important for veterinary medicine by the World Organisation for Animal Health (OIE), as they treat a wide range of diseases, including septicemias and urinary, respiratory, and digestive infections in livestock and pets^21^. Additionally, gentamicin is the main medication indicated for *Pseudomonas aeruginosa* infections, with few alternatives available for effective treatment^21^.

*ErmC*, which is categorized in the macrolide-lincosamide-streptogramin B resistance group (SI Table 2), can be induced by *Staphylococcus aureus*^39^. Erythromycin is an example of a macrolide drug to which *ermC* can provide resistance and is currently used in animals in Nepal (SI Table 1). As stated by the OIE, macrolides cover a wide range of diseases in animals, including those sampled in the current study (poultry and swine), with few alternative medications should macrolides fail^21^. The strain of β-lactamase for which this study tested (SHV(238G240E)) is linked to *E. coli* in GenBank (*E. coli* strain HB101 SHV-1 β-lactamase (*bla*_SHV-1_) gene^40^). Classified as a Class A β-lactamase (SI Table 2), *bla*_SHV-1_ is included in the genes that are the primary cause of resistance to beta-lactam drugs among Enterobacteriaceae, including cephalosporins and penicillins such as amoxicillin, ampicillin, Cloxacillin, benzylpenicillin (penicillin G), and phenoxymethylpenicillin (penicillin V), all in use in Nepal in animals^38^ (SI Table 1). Beta-lactams are extremely important for veterinary medicine as they are used for the treatment of septicemias as well as urinary and respiratory infections across a broad range of animal species with few economical alternatives currently available^21^. Quinolones such as nalidixic acid as well as fluoroquinolones such as ciprofloxacin are both targeted by the *QnrS* gene via diverse molecular mechanisms of resistance. Additionally, both drug classes are critically important in veterinary medicine in Nepal^21^. This is also true of the tetracycline drug class, with drugs such as doxycycline and tetracycline used to treat many species and a wide range of diseases^21^. Both *tetA* and *tetB* provide resistance to tetracyclines via encoding for proteins that act as tetracycline efflux pump proteins^41^.

The detection of these seven resistance genes found across animal populations in our study is of concern, as the genes could already be circulating in pathogens. Moreover, if the genes are not already found in pathogenic bacteria in the community, they might reside on mobile genetic elements or commensal bacteria as part of the community resistome and may be transferred to pathogenic bacteria in the future, thus providing a source of resistance to antibiotics that are currently in use in animals in Nepal that could spread within the community. Further, more targeted research should explore where these genes exist within the community resistome (e.g., in commensal or pathogenic bacteria or on mobile genetic elements). Additionally, we found these resistance genes in humans as well as animals and water in our study, and as the same drugs mentioned above are also indicated in humans in Nepal, the risk for AMR presence and transmission exists across populations studied.

### Importance in human medicine in Nepal

In this study, we prioritized antibiotic classes that are important for use in the human population in Nepal^38^ (SI Table 1). In addition to the OIE’s list of critically important antimicrobials for veterinary medicine, the World Health Organization (WHO) published and continually updates a list of priority pathogens with the goal of aiding in research and development of new antibiotics as well as supporting good antibiotic stewardship and furthering policies in both the human and veterinary/agricultural spheres^42^. Prior to the publication of this list, antibiotic research and development was driven by factors such as investor pressure, technological availability, and market size, and did not focus on crucial parameters such as global bacterial pathogens that have few alternative treatments and have shown an increase in resistance^42^. Because of the priority pathogen list’s publication, research and development priorities can now be better aligned with global health needs, thus supporting the fight against antibiotic resistance worldwide.

Resistance genes in this study are associated with pathogenic bacteria that are listed under all three priority levels set by the WHO in their priority pathogen list^42^ (SI Table 1). The highest priority (level 1, critical) focuses on carbapenem-resistant Enterobacteriaceae including *E. coli*, as well as *Pseudomonas aeruginosa* from non-human sources^42^. Diverse β-lactamases that confer this resistance, such as *CTX-M*, *TEM*, and *SHV* type genes (including *bla*_SHV-1_, the gene in the current study), have been observed in *E. coli, Salmonella* spp., *Klebsiella pneumoniae* and other Enterobacteriaceae from non-human sources such as livestock and companion animals^24,43–47^. High priority pathogens (level 2, high) include fluoroquinolone-resistant *Salmonella spp*., associated with *Qnr* resistance genes such as the one studied here, *QnrS*. A high prevalence of *Salmonella* has been documented in Nepal in humans, animals, and the environment, including in meat markets in Kathmandu^48,49^. Additionally, *QnrS* is associated with one of the medium priority pathogens on the WHO list (priority 3), fluoroquinolone-resistant *Shigella spp.*^42^.

Although antibiotic use has not been historically well-documented in humans in Nepal, it is acknowledged to be widespread and is inadequately regulated^50^. Additionally, prior research has found a high prevalence of resistant organisms in both hospital and community settings in Nepal^50^. The lax regulations on widespread antibiotic use in both human and animal populations allow for the transfer of resistance from the animal to human population or vice versa via both direct and indirect methods. Furthermore, with common antibiotics in use such as those listed in SI Table 1, it is essential that the necessity and usage of these drugs are better prioritized and managed in both human and animal populations to help limit the spread of resistance, thus allowing for their prolonged efficacy, as all are listed as highly or critically important in both populations.

While this study assessed prevalence of a broad range of resistance genes with public health significance, we did not simultaneously characterize commensal or pathogenic bacteria in the samples to inform on specific bacteria from which these genes have been identified to provide evidence for transmission mechanisms between human and animal populations. Future work involving 16S metagenome sequencing to identify bacteria in which the AMR genes reside would provide a more comprehensive overview of all antibiotic resistant genes and mobile genetic elements in this microbial community. Additionally, it is important to note that the presence of these resistance genes does not necessarily mean clinical treatment failure, because in vitro testing with minimum inhibitory concentrations (MIC) of corresponding microorganisms was not done. As resistance genes can be found on mobile genetic elements as well as in unculturable commensal bacteria, especially in the environment (not just in pathogenic bacteria), we focused on characterizing the genetic resistome of the community. Future research should expand on our findings with studies involving metagenomic and culture-based methods. Further work could also follow the framework set up by Martínez et al. (2015) to assess AMR risk in humans and animals based on carriage of resistance genes in this community^51^.

In summary, this study characterized and highlighted risk at the animal-human-environmental interface for the presence of seven resistance genes associated with antibiotics and resistant pathogens of public health importance in an urban informal settlement in Kathmandu, Nepal from a One Health perspective. These seven genes (*bla*_SHV-1_ (SHV(238G240E) strain), *QnrS*, *ermC*, *tetA*, *tetB, aacC2*, and *aadA1*) span a broad range of drug resistance classes, including aminoglycosides, beta-lactams, tetracyclines, macrolides, and fluoroquinolones. Animal, human, and environmental reservoirs for these genes may reside in the community as well as hospital settings, and by distinguishing parameters that increase the risk of finding a resistance gene, we can guide AMR policies in the larger community context from a one health perspective.

## Methods

### Study Setting

Concurrent sampling was undertaken in July 2017 at Jadibuti, an informal settlement site along the Manohara River in southeastern Kathmandu, Nepal. At the time of sampling, there were roughly 380 households and over 1,500 people living in the settlement, and backyard livestock production was prevalent in the area. The study site was part of USAID’s Emerging Pandemic Threats PREDICT program undertaking a one health surveillance strategy to strengthen capacity for zoonotic disease detection in 30 countries in Asia and Africa^52^. All sampling was conducted by a team trained and supervised by the Center for Molecular Dynamics Nepal (CMDN). Ethical approval was obtained from the National Health Research Council in Nepal (NHRC, Ref. 1438) and the UC Davis IRB, under the PREDICT Master IRB protocol (804,522–3). All sampling was performed in accordance with relevant guidelines and regulations for both humans and animals, and followed the recommendations in the ARRIVE guidelines^53^.

The target sample size following informed consent and screening procedures was 205 total samples, which assumed an expected proportion of 10% having AMR genes, a population size of 1,618 (n = 381 households in Jadibuti and an average of 10 humans and animals per household), and an expected response rate of 90%. A design effect (the variance accounting for clustered sampling divided by the variance assuming a simple random sample) was added to the sample size calculation to adjust for relatedness of samples within households. The design effect was set at 2, assuming the variance was twice as large as would be expected with simple random sampling^54^. A total of 218 specimens from 161 individuals were tested across 35 households, including: humans (n = 88), chickens (n = 47), ducks (n = 35), swine (n = 11), and water (n = 17) clustered by household (n = 35), as well as rodents (n = 8) and shrews (n = 12) captured near dwellings.

### Sample Collection

Participant households were selected using a simple random sampling technique. Enumerators aimed to sample at least two individuals and at least two pairs of owned animals (e.g., two swine and two ducks) per household. The following samples were collected: oral samples (oropharyngeal swabs from ducks, chickens, and humans); fecal samples (rectal/cloacal for swine, ducks, chickens, rats, and shrews, and self-collected stool specimens from humans); and water samples (from household storage, wells, and the river using vacuum filtration [Millipore Co., Billerica, MA]). Informed consent was obtained from all participants, or from a parent/legal guardian if participants were under 18. After obtaining consent, participants who satisfied inclusion criteria were asked to provide two oral samples and a stool specimen. Oral and fecal samples were also collected from: chickens, ducks, swine, rodents (*Rattus spp.*), and shrews (*Suncus murinus, Crocidura lasiura, Soricidae*). Duplicate water samples were collected for households sampled as well as the adjacent river and several community wells, with raw water used for fecal indicator testing performed using an Aquagenx kit, following standard kit protocol (Aquagenx, Chapel Hill, North Carolina, USA). All samples were labeled by household using a unique, anonymous ID. Samples were immediately put on ice and transferred to the laboratory within 8 h, for storage until analysis.

QIAamp DNA Stool Mini Kits were used to extract DNA from human fecal samples, and QIAamp UCP Pathogen Mini Kits were used for oral, cloacal, and rectal swabs (QIAGEN, Valencia, California, USA). DNA from water sample filters was extracted using the DNeasy PowerWater Kit (QIAGEN, Valencia, California, USA). Eluted DNA was shipped to the University of California, Davis. An Applied Biosystems 7500 Fast machine (Applied Biosystems, Waltham, MA, USA) was used to perform real-time qualitative PCR (qPCR) with the Antibiotic Resistance Genes array, a microbial DNA qPCR array containing assays for 16 antibiotic resistance classification groups (QIAGEN, Valencia CA). Each array contained two host assays (GAPDH, HBB1) to detect the presence of host (human/mouse) genomic DNA, two pan-bacteria assays (PanB1, PanB2) designed to detect a broad range of bacterial species, as well as a positive PCR control (PPC) to test for the presence of inhibitors in the sample as described (https://www.qiagen.com/us/products/discovery-and-translational-research/pcr-qpcr-dpcr/qpcr-assays-and-instruments/microbial-dna-qpcr-assays-and-panels/microbial-dna-qpcr-arrays/).

Following QIAGEN protocol, baseline cycle (8–20 cycles) and threshold (0.2) settings were manually applied for each run. Raw Ct values were exported to excel for use with QIAGEN’s Microbial DNA qPCR Array Data Analysis Template Excel Software, available online from: http://www.qiagen.com/Products/Catalog/Assay-Technologies/Real-Time- PCR-and-RT-PCR-Reagents/Microbial-DNA-qPCR-Array. The presence or absence of a resistance gene was identified using a ∆C_T_ method in which the C_T_ value is inversely correlated with the abundance of genes in the sample. This method used a no template control (NTC) array to determine background contamination of the array, thereby allowing for higher sensitivity of detection of genes. Lower and upper C_T_ values for positive and negative results, respectively, were established using the NTC. For this analysis, the upper limit for a positive result was 34 (NTC-6) and the lower limit for a negative result was 37 (NTC-3). Values in between 34 and 37 were considered inconclusive by the software and thus were conservatively recoded as negative.

### Genes of Importance for Analyses

Resistance genes were identified and resistance gene prevalences were calculated for every household and for humans and animals sampled in each household. Genes of public health importance were prioritized for this study based on the WHO’s lists of AMR priority pathogens and of critically important antibiotics for human and animal health, as well as the prevalence of the gene in the community based on sample detection. After evaluating all 88 resistance genes, we chose the following seven genes based on sample prevalence: *bla*_SHV-1_ (SHV(238G240E) strain), *QnrS*, *ermC*, *tetA*, *tetB, aacC2*, and *aadA1*.

*bla*_SHV-1_ is a Class A β-lactamase resistance gene (SHV(238G240E), accession # AF148850) that was originally isolated in the 1970s from *E. coli*^55^. Carbapenem-resistant, ESBL-producing *Enterobacteriaceae* are currently on the WHO’s priority pathogens list for research and development of new antibiotics (priority 1: critical)^1^.

*QnrS* (accession # AB187515) confers fluoroquinolone resistance, and the QIAGEN assay may also detect *QnrS1*, *QnrS2*, *QnrS3*, or *QnrS4*. *QnrS* has been found in the following genera of bacteria: *Aeromonas*, *Enterobacter, Escherichia, Klebsiella, Proteus, Salmonella, Shigella, Vibrio,* and *Vibrionales*^41^. Additionally, *Qnr* genes have been found globally in enterobacterial species, and are reported as associated with mobile genetic elements^56^.

*ermC* (accession # Y09002.1) confers resistance to erythromycin and has been found in the following genera: *Bacillus, Lactobacillus, Neisseria,* and *Staphylococcus*^41^. QIAGEN classifies it in its macrolide-lincosamide-streptogramin B classification group.

*tetA* and *tetB* (accession #s AY196695.1 and AF223162.1, respectively) provide resistance against tetracycline, and have been found in a plethora of bacterial genera, including: *Acinetobacter, Aeromonas, Escherichia, Neisseria, Klebsiella, Pseudomonas, Salmonella, Shigella, Streptococcus, Staphylococcus, Vibrio*, and *Yersinia*^41,57^.

*aacC2* (accession # EU022314), synonymous with AAC(3)-IIc, is plasmid-encoded and found in *E. coli* and *P. aeruginosa*^58^. This gene modifies aminoglycosides by acetylation^41,59^.

*aadA1* (accession # DQ370505) confers streptomycin and spectinomycin resistance^60^. It is an aminoglycoside nucleotidyltransferase gene that is encoded by plasmids, transposons, and integrons, and is found in Enterobacteriaceae, *A. baumannii*, *P. aeruginosa, Vibrio cholera,* and *E. coli*^58,60^.

### Statistical Analyses

To assess which factors influenced the presence of these genes in the community, we performed multilevel logistic regression using the lme4 R package^61^ for each resistance gene chosen with the outcome as presence/absence of the gene in the sample tested. Fixed effect variables in each full model included: specimen sample type (categorical: both fecal and oral, oral swab, rectal swab, feces, well, jar, river), specimen sample source (categorical: swine, duck, chicken, rodent, shrew, human, water), and how many people lived in the dwelling in which the specimen was collected (numeric), with zero recorded for sources sampled that were not connected to a household (rodents, shrews, and river water), and co-occurrence of genes in a sample, as measured by the total number of genes positive (out of 88 possible) in the specimen sample (numeric).

To account for a potential household effect (samples might be more similar by household), hierarchical mixed effects models were used with household ID as a random effect. Evidence for clustering was assessed via the household ID random effect variance, the reported intraclass cluster coefficient (ICC)^62^, and graphic results of variance across households sampled. Models with a random effect variance and an ICC of 0 (evidence of clustering by household was not identified) were simplified to fixed effects logistic regression models. A subset of data (n = 161) was used in these analyses, where within-individual samples were collapsed and counted as positive for both oral and fecal sampling if both of the two samples (fecal or oral) were positive for a particular resistance gene, and otherwise categorized according to whether the oral or fecal sample was positive. Bivariate analyses of the outcome and each predictor variable were conducted, with a conservative cutoff of p < 0.20 for further evaluation in the regression models. Predictor variables that were not significant (p < 0.05) were dropped from each full model. Model diagnostics were performed on each final model to assess goodness-of-fit and to check model assumptions. Deviance residuals and AICs were used to compare model fit when constructing a final model for each gene. Odds ratios (ORs) and 95% confidence intervals (CIs) were reported for each final model. All statistical analyses were conducted in R Studio, Version 1.1.453^63^.

## Supplementary Information

Supplementary Information.

## Data Availability

Data analyzed are published with manuscript.
