# Supplementary material for: Antibiotic resistance genes of public health importance in livestock and humans in an informal urban community in Nepal
Source: Sci Rep. 2022 Aug 15;12:13808. doi: 10.1038/s41598-022-14781-y (PMC9378709; doi:10.1038/s41598-022-14781-y)
Supplement: Supplementary file 1 — Supplementary Information. [file 41598_2022_14781_MOESM1_ESM.docx]

**SI Table 1. Antibiotic resistance genes and examples of antibiotics used in humans and animals in Nepal with WHO and OIE medical importance priority rankings and comments**

| **Gene** | **Antibiotic Classification Group** | **Example antibiotic*** | **WHO ranking**** | **OIE ranking***** | **Animal species in which antibiotic is used****** | **OE and WHO comments** |
| --- | --- | --- | --- | --- | --- | --- |
| *bla_SHV-1_* (SHV(238G240E) strain) | Class A β-lactamase | Amoxicillin | CI | VCIA | Avian, bovine, caprine, equine, ovine, fish, swine | WHO: May result from transmission of *Enterococcus* spp., Enterobacteriaceae, including *E. coli*, as well as *Pseudomonas* *aeruginosa* from non-human sources |
|  |  | Ampicillin | CI | VCIA | Avian, bovine, caprine, equine, ovine, fish, swine | WHO: May result from transmission of *Enterococcus* spp., Enterobacteriaceae, including *E. coli*, as well as *Pseudomonas* *aeruginosa* from non-human sources |
|  |  | Cloxacillin | HI | VCIA | Bovine, caprine, equine, ovine, swine | WHO: In certain geographic settings, the class may be one of limited therapies for staphylococcal infections (*S. aureus*); may result from transmission of *S. aureus*, including MRSA, from non-human sources |
|  |  | Benzylpenicillin (penicillin G) | CI | VCIA | Bovine, camel, caprine, equine, ovine, swine | WHO: May result from transmission of *Enterococcus* spp., Enterobacteriaceae, including *E. coli*, as well as *Pseudomonas* *aeruginosa* from non-human sources |
|  |  | Phenoxymethylpenicillin (penicillin V) | CI | VCIA | Avian, swine | WHO: May result from transmission of *Enterococcus* spp., Enterobacteriaceae, including *E. coli*, as well as *Pseudomonas* *aeruginosa* from non-human sources |
| *QnrS* | Fluoroquinolone resistance | Ciprofloxacin | CI | VCIA | Avian, bovine, swine | WHO: Limited therapy for *Campylobacter* spp., invasive disease due to Salmonella, and MDR *Shigella* spp. Infections; may result from transmission of *Campylobacter* spp. and Enterobacteriaceae, including *E. coli* and *Salmonella* from non-human sources |
|  |  | Nalidixic acid | CI | VHIA | Bovine | WHO: Limited therapy for *Campylobacter* spp., invasive disease due to Salmonella, and MDR *Shigella* spp. Infections; may result from transmission of *Campylobacter* spp. and Enterobacteriaceae, including *E. coli* and *Salmonella* from non-human sources |
| *ermC* | Macrolide-Lincosamide-Streptogramin B | Azithromycin | CI | - | - | WHO: Limited therapy for *Legionella*, *Campylobacter*, and MDR *Salmonella* and *Shigella* infections; may result from transmission of *Campylobacter* spp. and *Salmonella* from non-human sources |
|  |  | Erythromycin | CI | VCIA | Bee, avian, bovine, caprine, equine, rabbit, ovine, fish, swine | WHO: Limited therapy for *Legionella*, *Campylobacter*, and MDR *Salmonella* and *Shigella* infections; may result from transmission of *Campylobacter* spp. and *Salmonella* from non-human sources |
| *tetA, tetB* | Tetracycline efflux pump | Doxycycline | HI | VCIA | Avian, bovine, camel, caprine, equine, rabbit, ovine, fish, swine | WHO: Limited therapy for infections due to *Brucella* spp., *Chlamydia* spp., and *Rickettsia* spp; countries where transmission of brucellosis from non-human sources to humans is common should consider making tetracycline a critical antibiotic, as there is considerable concern regarding the availability of effective products where *Brucella* spp. are endemic |
|  |  | Tetracycline | HI | VCIA | Bee, avian, bovine, camel, caprine, equine, rabbit, ovine, fish, swine | WHO: Limited therapy for infections due to *Brucella* spp., *Chlamydia* spp., and *Rickettsia* spp; countries where transmission of brucellosis from non-human sources to humans is common should consider making tetracycline a critical antibiotic, as there is considerable concern regarding the availability of effective products where *Brucella* spp. are endemic |
| *aacC2, aadA1* | Aminoglycoside-resistance | Gentamicin | CI | VCIA | Avian, bovine, camel, caprine, equine, rabbit, ovine, swine | OIE: Indicated for *Pseudomonas* *aeruginosa* infections with few alternatives  WHO: Sole or limited therapy as part of treatment of enterococcal endocarditis and multidrug resistant (MDR) tuberculosis; may result from transmission of *Enterococcus* spp., Enterobacteriaceae (including *E. coli*), and *Mycobacterium* spp. from non-human sources |
|  |  | Streptomycin | CI | VCIA | Bee, avian, bovine, caprine, equine, rabbit, ovine, fish, swine | WHO: Sole or limited therapy as part of treatment of enterococcal endocarditis and multidrug resistant (MDR) tuberculosis; may result from transmission of *Enterococcus* spp., Enterobacteriaceae (including *E. coli*), and *Mycobacterium* spp. from non-human sources |

*Example of antibiotic that is currently used in both animals and humans in Nepal^38^

**WHO ranking for use in both humans and animals; CI: critically important; HI: highly important^3^

***OIE ranking for use in animals; VCIA: veterinary critically important antibiotic agent; VHIA; veterinary highly important antibiotic agent^21^

****from the OIE ^21^

**SI Table 2. Antibiotic classification category and antibiotic resistance genes in the QIAGEN Antibiotic Resistance Genes Microbial DNA qPCR Array**

| **Antibiotic Classification/Description** | **Antibiotic Resistance Gene*** |
| --- | --- |
| Fluoroquinolone resistance | *AAC(6’)-Ib-cr* |
| Aminoglycoside resistance | *aacC1* |
|  | ***aacC2*** |
|  | *aacC4* |
|  | ***aadA1*** |
|  | *aphA6* |
| Class A β-lactamase | *BES-1* |
|  | *BIC-1* |
|  | *CTX-M-1* Group |
|  | *CTX-M-8* Group |
|  | *CTX-M-9* Group |
|  | *GES* |
|  | *IMI & NMC-A* |
|  | *KPC* |
|  | *Per-1* group |
|  | *Per-2* group |
|  | *SFC-1* |
|  | *SFO-1* |
|  | *SHV* |
|  | *SHV(156D)* |
|  | *SHV(156G)* |
|  | ***SHV(238G240E)*** |
|  | *SHV(238G240K)* |
|  | *SHV(238S240E)* |
|  | *SHV(238S240K)* |
|  | *SME* |
|  | *TLA-1* |
|  | *VEB* |
| Class B β-lactamase | *ccrA* |
|  | *IMP-1* group |
|  | *IMP-12* group |
|  | *IMP-2* group |
|  | *IMP-5* group |
|  | *NDM* |
|  | *VIM-1* group |
|  | *VIM-13* |
|  | *VIM-7* |
| Class C β-lactamase | *ACC-1* group |
|  | *ACC-3* |
|  | *ACT 5/7* group |
|  | *ACT-1* group |
|  | *CFE-1* |
|  | *CMY-10* Group |
|  | *DHA* |
|  | *FOX* |
|  | *LAT* |
|  | *MIR* |
|  | *MOX* |
| Class D β-lactamase | *OXA-10* Group |
|  | *OXA-18* |
|  | *OXA-2* Group |
|  | *OXA-23* Group |
|  | *OXA-24* Group |
|  | *OXA-45* |
|  | *OXA-48* Group |
|  | *OXA-50* Group |
|  | *OXA-51* Group |
|  | *OXA-54* |
|  | *OXA-55* |
|  | *OXA-58* Group |
|  | *OXA-60* |
| Erythromycin resistance | *ereB* |
| Fluoroquinolone resistance | *QepA* |
|  | *QnrA* |
|  | *QnrB-1* group |
|  | *QnrB-31* group |
|  | *QnrB-4* group |
|  | *QnrB-5* group |
|  | *QnrB-8* group |
|  | *QnrC* |
|  | *QnrD* |
|  | ***QnrS*** |
| Macrolide-Lincosamide-Streptogramin B | *ermA* |
|  | *ermB* |
|  | ***ermC*** |
|  | *mefA* |
|  | *msrA* |
| Multidrug resistance efflux pump | *oprj* |
|  | *oprm* |
| Tetracycline efflux pump | ***tetA*** |
|  | ***tetB*** |
| Vancomycin resistance | *vanB* |
|  | *vanC* |
| Staphylococcus aureus | *Staphylococcus aureus* |
| Beta-lactam resistance | *mecA* |
| Panton-Valentine leukocidin NPACain F precursor | *lukF* |
| Immunoglobulin G binding protein A precursor | *spa* |
| Methicillin Resistant Staphylococcus aureus | *MRSA* |

*Bolded genes were evaluated in this analysis.
